# Tight Interconnection and Multi-Level Control of Arabidopsis MYB44 in MAPK Cascade Signalling

**DOI:** 10.1371/journal.pone.0057547

**Published:** 2013-02-21

**Authors:** Helene Persak, Andrea Pitzschke

**Affiliations:** Department of Applied Genetics and Cell Biology, University of Natural Resources and Life Sciences, Vienna, Austria; Ghent University, Belgium

## Abstract

Abiotic stress poses a huge, ever-increasing problem to plants and agriculture. The dissection of signalling pathways mediating stress tolerance is a prerequisite to develop more resistant plant species. Mitogen-activated protein kinase (MAPK) cascades are universal signalling modules. In Arabidopsis, the MAPK MPK3 and its upstream regulator MAPK kinase MKK4 initiate the adaptation response to numerous abiotic and biotic stresses. Yet, molecular steps directly linked with MKK4 – MPK3 activation are largely unknown. Starting with a yeast-two-hybrid screen for interacting partners of MKK4, we identified a transcription factor, MYB44. MYB44 is controlled at multiple levels by and strongly inter-connected with MAPK signalling. As we had shown earlier, stress-induced expression of the *MYB44* gene is regulated by a MPK3-targeted bZIP transcription factor VIP1. At the protein level, MYB44 interacts with MPK3 *in vivo.* MYB44 is phosphorylated by MPK3 *in vitro* at a single residue, Ser145. Although replacement of Ser145 by a non-phosphorylatable (S145A) or phosphomimetic (S145D) residue did not alter MYB44 subcellular localisation, dimerization behaviour nor DNA-binding characteristics, abiotic stress tolerance tests in stable transgenic Arabidopsis plants clearly related S145 phosphorylation to MYB44 function: Compared to Arabidopsis wild type plants, *MYB44* overexpressing lines exhibit an enhanced tolerance to osmotic stress and are slightly more sensitive to abscisic acid. Interestingly, overexpression of the S145A variant revealed that impaired phosphorylation does *not* render the MYB44 protein non-functional. Instead, S145A lines are highly sensitive to abiotic stress, and thereby remarkably similar to *mpk3*-deficient plants. Its *in vivo* interaction with the nuclear sub-pools of both MPK3 and MKK4 renders MYB44 the first plant transcription factor to have a second function as putative MAPK cascade scaffolding protein.

## Introduction

Environmental stresses represent the most limiting factors for agricultural productivity worldwide. Climate change and the unpredictable onset of abiotic or biotic adversities are a major challenge to any living species. As plants cannot escape environmental threats, they need to adapt at the cellular and physiological level to withstand unfavourable cues. A major mechanism linking environmental stress perception to cellular responses involves signalling through Mitogen-activated protein kinase (MAPK) cascades [1], [2].

MAPK cascades are eukaryotic signalling modules that consist as a minimum of a MAPK kinase kinase (MAPKKK), a MAPKK and a MAPK, which via a phospho-relay system serve both signal transduction and amplification. By altering phosphorylation-dependent properties of their target proteins, activated MAPKs translate the information further, eventually leading to e.g. changes in gene expression, cellular redox state or cell integrity. In contrast to animals, where MAPK cascades have been studied extensively and several MAPK substrates been identified (reviewed in [3]), comparatively little is known about individual MAPK cascades and their direct targets in plants [4], [5].

### 1200 combinations and the role of scaffolding proteins

The *Arabidopsis thaliana* genome encodes for 60 MAPKKKs, 10 MAPKKs and 20 MAPKs [6]. Similar family sizes are found in other plant species. Specificity, *i.e.* which of the theoretically possible 60×20×10 = 1200 module combinations is formed and integer under a certain condition, can be conferred to by specific expression or subcellular localisation of MAPK module components, or through the action of scaffold proteins, shared docking domains, and adaptor or anchoring proteins [7]–[9]. Scaffolding proteins have been shown to prevent cross talks between MAPK pathways employing a common MAPKKK in yeast, and also in mammalian cells [10], [11].

The MAPKKs MKK4 and its closest homolog, MKK5, form one of the four phylogenetic groups of MAPKKs in Arabidopsis [6]. In protoplasts, treatment with the bacterial elicitor flagellin, initiates the cascade MAPKKK MEKK1 MKK4/MKK5 MPK3 and MPK6 [12], followed by the induction of stress-responsive genes. The same module [MEKK1 – MKK4/5 – MPK3/6] functions in the response to fungal elicitors, where it regulates the synthesis of camalexin, a major antimicrobial compound [13]. An alternative cascade, comprising the MAPKKK Yoda – MKK4/MKK5 – MPK3/MPK6, is a key regulator of stomatal development [14]. Induction of constitutively active variants of MKK4/MKK5 or their closest homolog in tobacco, NtMKK2, initiates the strong and permanent activation of the corresponding MAPKs, followed by cell death after 24 h [13], [15]. MPK3 and MPK6 have largely, but not entirely, overlapping functions [5], [14], [16], [17]. Mutants deficient in both MAPKs are embryo-lethal [14].

### Transcription factors

MAPK-mediated stress responses to environmental cues will ultimately involve major changes at the transcript, protein and metabolic level. Since MAPK(KK)s themselves normally have no DNA-binding activity, re-programming of gene expression can be accomplished through phosphorylation of transcription factors, thereby altering protein properties such as subcellular localisation, protein stability, homo/heterodimerisation, trans-activating capacity, target DNA motif affinity and –specificity [18]–[22].

A small number of transcription factors associated with MAPK signalling have been identified and functionally characterised, including primarily members of the WRKY family [23], [24]. MPK3 and MPK6 enhance the stability of AtEIN3 [25] and the DNA-binding affinity of AtERF104 [26], two transcription factors involved in ethylene signalling. Phosphorylation by MPK3 regulates the subcellular localisation of the bZIP protein VIP1 in a stress-dependent manner [18].

MYB proteins represent another large family of plant transcription factors, comprising over 120 members in Arabidopsis [27]. MYB proteins regulate transcription by binding to their target motifs as MYB/MYB homo- or heterodimers. Examples also exist for regulatory complexes formed by MYB proteins and other types of transcription factors [27], [28]. Members of the MYB family have been characterised to various extent in a diversity of species, and a role in germination, development, and responses to stress and hormone treatment could be [27], [28]. Interspecies comparisons revealed a good correlation between sequence homology and function in some cases. For example, involvement of MYBs in the regulation of anthocyan synthesis appears to be restricted to a fraction of closely related family members [27], [29]. However, MYB function in stress responses cannot be ascribed to a particular subfamily [27], [29]. PtMYB4, a MYB protein with a putative function in xylem development, is phosphorylated by PtMPK6 *in vitro*, and studies in yeast suggest that phosphorylation promotes its trans-activating capacity [21]. Although several MYB proteins were among putative MAPK targets, isolated from a phosphopeptide screen [30], *in vivo* evidence of individual MAPK – MYB relations is still very limited. Given the documented role of MAPK cascades as stress mediators and the involvement of plant MYB proteins in diverse abiotic [29] and biotic [27] stress responses, a MAPK-regulated control of MYB transcription factors, similar to those of the WRKY family, appears likely.

Our earlier studies had indirectly linked a multiple-stress-responsive Arabidopsis *MYB* gene to MAPK-mediated stress signalling [18], [31].Specifically, upon stress treatment, MPK3 becomes rapidly activated and phosphorylates the bZIP transcription factor VIP1 (virE2-interacting protein 1). Subsequently, phosphorylated VIP1 translocates to the nucleus, where it induces the expression of early stress-responsive genes, including *MYB44* via direct promoter binding [31].

Here, we characterise MYB44 as direct and immediate component in the MKK4 – MPK3 signalling pathway. We locate the MPK3-phosphorylated residue in MYB44 and assess the effect of phosphorylation on MYB44 protein properties and its impact on abiotic stress tolerance. The role of MPK3 as post-translational regulator of MYB44 adds to its previously documented involvement in transcriptional regulation of the *MYB44* gene. I*n vivo* interaction of the MYB44 transcription factor with the nuclear subpools of MAPK cascade components is indicative of a second function, as scaffolding protein of the MKK4 – MPK3 module.

## Results

### Formation of MYB44 – MKK4 complexes and MYB44 homodimers in yeast

With a prime interest in signalling proteins associated with the MKK4 – MPK3 submodule, we employed the yeast-two-hybrid (Y2H) system to screen an Arabidopsis cDNA library, using MKK4 as bait.

**Figure 1:**
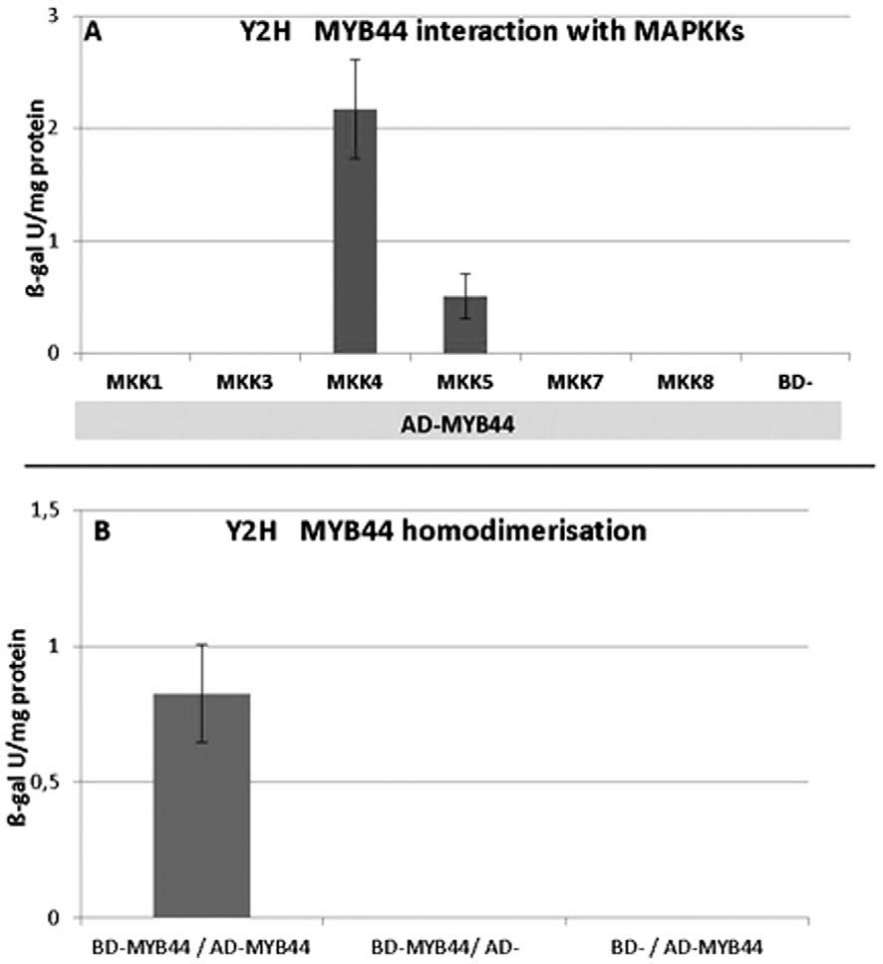
Yeast-two-hybrid studies on MYB44. A) MYB44 interacts with MKK4 in yeast. b-galactosidase activity in protein extracts from yeast co-transformed with activation-domain (AD) fusions to MYB44 and binding domain (BD) fusions to representative members of MAPKK family. Values of MKK autoactivity were substracted. Specific interaction of MYB44 with MKK4, and weakly with MKK5, was detected. B) MYB44 forms homodimers in yeast. ß-galactosidase activity in protein extracts form yeast co-transformed with BD-MYB44 and AD-MYB44 or with the the corresponding empty-vector controls.

MKK4 interaction was assessed by yeast growth on selective medium and subsequent liquid ß-galactosidase assays. One of the interacting candidates was identified as transcription factor MYB44. Targeted yeast-two-hybrid analyses confirmed the interaction of (activation domain) AD-MYB44 and (binding domain) BD-MKK4 (**figure 1A**). The strength of interaction was comparable to that of a positive control, (BD-MKK4/AD-MPK6). No ß-galactosidase activity was found in extracts from yeast co-transformed with BD-MKK4 and the pGAD control; or AD-MYB44 and a BD-control construct. In contrast, the reporter gene was induced upon co-expression of BD-MYB44 / AD-MYB44, clearly indicative of MYB44 intraspecific complex formation (**figure 1B**).

Next, we tested via Y2H whether MYB44 would also interact with MPK3, the specific target of MKK4 in MAPK signalling. However, there was no specific GAL4 reporter gene activity in cells co-expressing MYB44 and MPK3. Likewise, co-transformation of MYB44 with the closest MPK3 homolog, MPK6, or other representative members of Arabidopsis MAPK subfamilies, yielded no detectable interaction (**figure S1**). These findings might suggest that MYB44 and MPK3 are no direct interacting partners, or could be indicative of poor protein interaction caused by interfering yeast proteins or by conformational constrains of the AD/BD fusion peptides. We therefore initiated alternative approaches in which we tested for regulatory crosstalk between MPK3 and MYB44.

### MPK3 phoshorylates MYB44 *in vitro*

**Figure 2:**
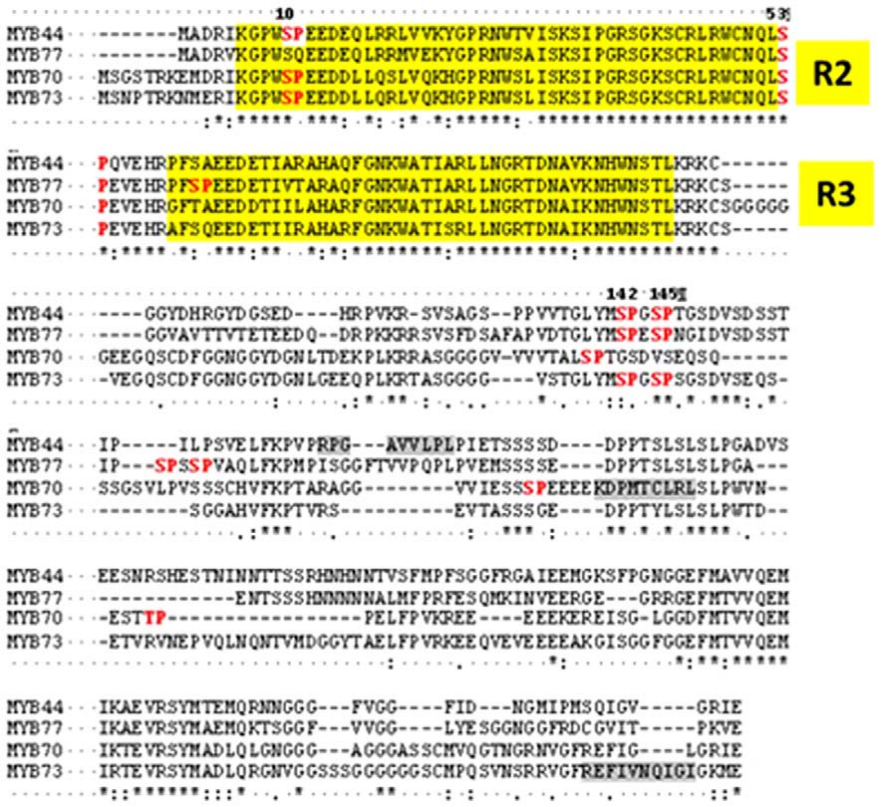
Protein alignment of MYB subfamily S22. The R2R3 repeats of the DNA-binding domain are indicated by yellow boxes. Ser-Pro dipeptides matching the minimal MAPK phosphorylation consensus motif (red) or putative MAPK docking sites (R/K x2-6 I/L×I/L) (grey) are highlighted. MYB44 Ser142 and Ser145 are indicated. *conserved residues.

Several MAPK interacting proteins from animals, yeast and also plants, carry a characteristic domain, termed “ KIM” (kinase interaction motif; R/K x2-6 I/L×I/L) [32]. Mutation analyses have shown an intact KIM to be required for Arabidopsis MAPK / phosphatase interaction [33]. Presence of KIM is an indication of but no absolute prerequisite for MAPK interaction. For instance, the MPK3 target protein VIP1 lacks such motif [18] . In MYB44, a putative KIM is located outside the MYB domain, in a non-conserved region (residues 171 to 179). Also, the protein sequence contains several sites matching the minimal consensus motif for MAPK-mediated phosphorylation, Ser-Pro or Thr-Pro (**figure 2**).

**Figure 3:**
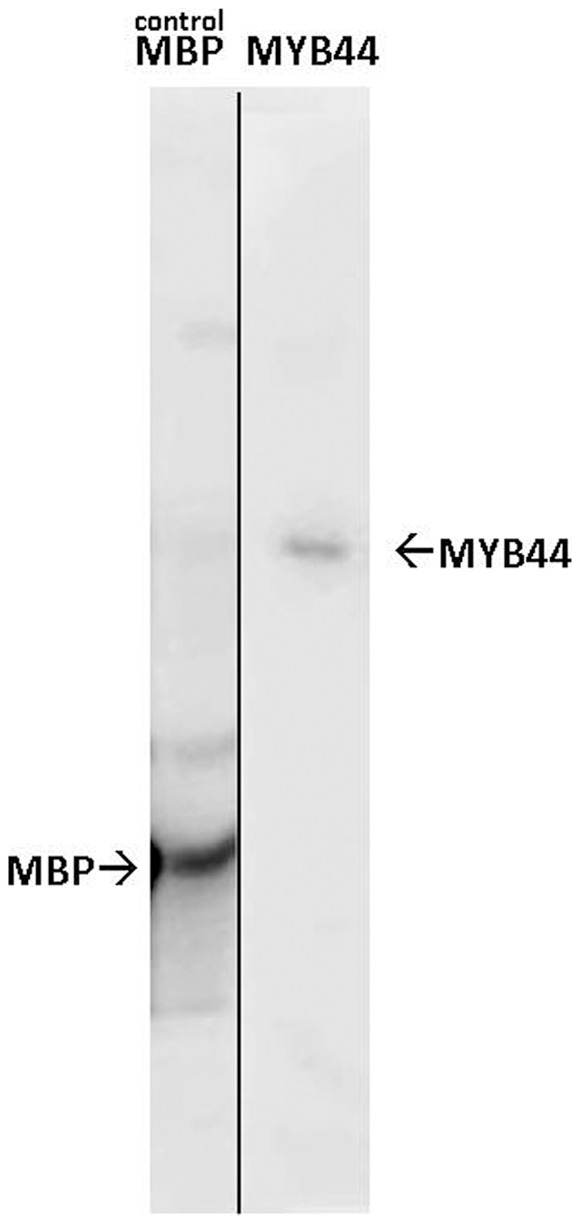
MYB44 is a MPK3 substrate. Purified non-tagged MYB44 recombinant protein was incubated with MPK3-GST in the presence of y32P-ATP. Phosphorylation was detected by autoradiography. The known MAPK substrate, myelin basic protein (MBP) was used to document MPK3 functionality.

*In vitro* kinase assays were performed to test whether MYB44 can be phosphorylated by MPK3. In order to circumvent possible inhibitory/artificial effects of fusion peptides on MYB44 accessibility for the kinase, we produced tag-free MYB44 recombinant protein, applying the intein-based strategy (NEB). MPK3 was produced as GST fusion protein, because strong kinase activity of recombinant GST-MPK3 is well-documented [18], [34]. The general MAPK substrate myelin basic protein (MBP) served as positive control. Both MBP and MYB44 were phosphorylated by MPK3. These findings indicate that MYB44 and MPK3 interact *in vitro*, and that one or several residues in MYB44 are targeted by the kinase (**figure 3**). MPK3/MYB44 complex formation was further supported by in vitro pull-down experiments (**figure S2**)

### MYB44 is phosphorylated by MPK3 specifically at Ser145

**Figure 4:**
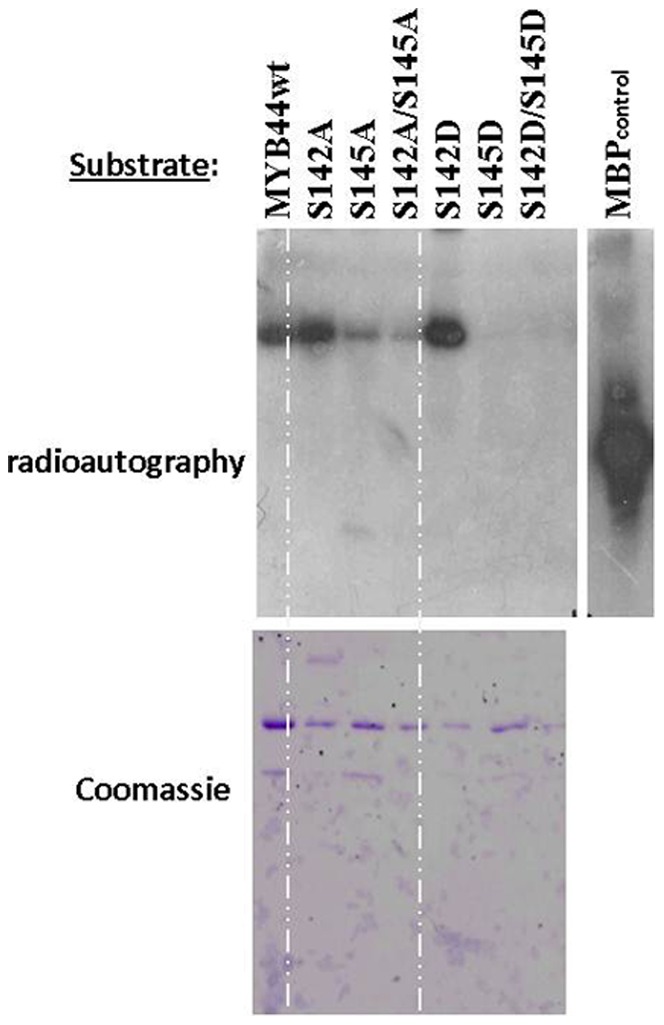
MPK3 phosphorylates MYB44 at residue Ser145. Recombinant proteins of MYB44 and Ser142/Ser145 mutated variants were incubated with MPK3-GST in the presence of y32P-ATP. Phosphorylation was detected by radioautography (top). The positive-control-substrate myelin basic protein (MBP) documents MPK3 functionality. Protein loading was visualised by Coomasie blue staining.

MYB44 contains four putative MAPK phosphorylation sites, located at S10, S53, S142 and S145 (**figure 2**). S10 and S53 are positioned in and between the R2/R3 repeats of the MYB DNA binding domain, respectively, whereas S142, S145 reside in a region close to the putative KIM (R171-L179). *In silico* analysis (EXPASY peptide cutter) predicts trypsin to cut at MYB44 residue: 4 6 18 19 23 27 34 39 42 45 47 71 79 85 90 96 104 105 106 113 125 126 209 223 240 248 266 270 278 and 303. The proximity of residues S142 / S145 and their expected occurrence on a common, comparatively large peptide (spanning residues 126–208), would impede reliable prediction of phosphorylated residue(s) by mass spectrometry. However, phylogenetic considerations helped to narrow down the number of candidate MYB44 phosphorylation target sites. MYB44, together with MYB70, MYB77 and MYB73 forms the S22 subfamily of R2R3MYB transcription factors [27], [35]. MYB44 residues S10 and S53 lie within the highly conserved region comprising the R2/R3 DNA-binding domain. All members of the S22 subfamily contain a serine residue corresponding to MYB44 S53, whereas a S10-corresponding site is found in MYB44, MYB70 and MYB73, but absent in MYB77. Comparison between MYB44, MYB70 and MYB77 with respect to their phosphorylatability may therefore help to restrict the number of MYB44 candidate phosphorylation sites. *In vitro* kinase assays with MYB44, MYB77 and MYB70 revealed MPK3-mediated phosphorylation of MYB44 and MYB77 but not of MYB70. These findings suggest a) that MYB77 is also a MPK3 target and b) that MYB44 residues S142 and/or S145, rather than S10 or S53 are the most likely targets sites for MPK3 phosphorylation (**figure S3**). Consequently, MYB44 variants mutated at residues Ser142 and/or Ser145 were generated. The exchange of serine residues to alanine (A) or aspartate (D) is a common approach for creating non-phosphorylatable or phosphomimetic variants, respectively (e.g. [18], [36]. Subsequent *in vitro* kinase assays revealed strong MPK3-mediated ^P^32P incorporation in MYB44 wild type protein as well as in the S142A and S142D variants. Contrary, signals were drastically reduced in S145A and [S142A/1455A]; and not detectable in S145D and [S142D/1455D] variants, respectively (**figure 4**), revealing S145 as major target site. The presence of strong signals in S142A and S142D variants, but complete absence in the phosphomimetic S145D variant furthermore indicates that S145 phosphorylation is not a prerequisite for subsequent phosphorylation at any of the remaining (S10, S53 or S142) residues. Thus, MPK3 targets only one of the four candidate sites; namely S145.

### Analyses of interaction between MYB44, MPK3 and MKK4 *in vivo*

**Figure 5:**
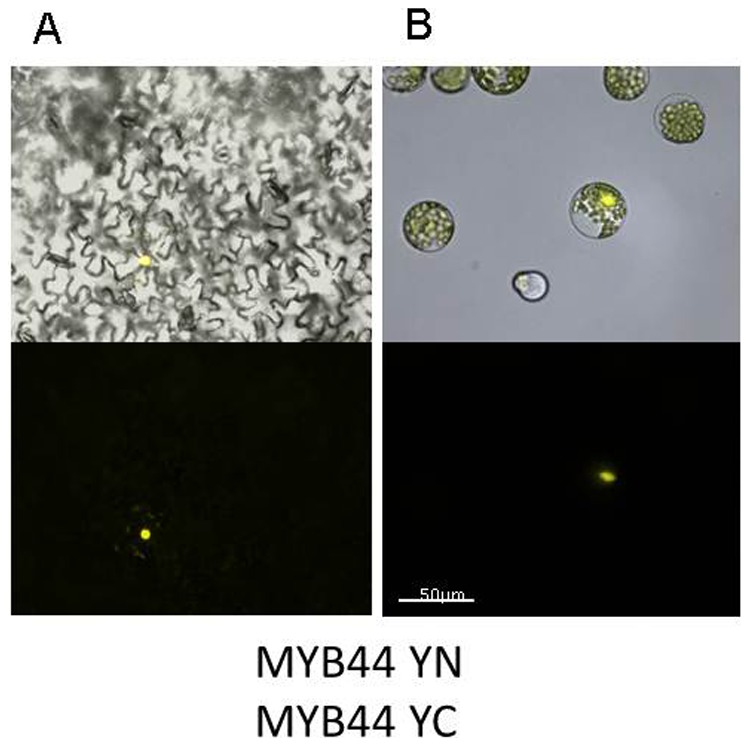
MYB44 homodimerisation *in planta.* Plant cells were co-transformed with MYB44 fused to the N- and C-terminal YFP region. (MYB44-YC/ MYB44-YN). Complex formation was observed in (A) tobacco leaves after 5 days and (B) Arabidopsis mesophyll protoplasts 16 h post-transformation by fluorescence microscopy. top line: brightfield/UV overlay; bottom line: UV image

Many transcription factors are known to form functional complexes. For example, some NAC and MADS proteins engage in homo- or hetero-dimeric or tetrameric aggregates [37]–[39]. Until now, the dimerization behaviour of MYB44 is unknown. In Y2H experiments, we had observed MYB44 / MYB44 interaction (**figure 1B**). Using bimolecular fluorescence (BiFC; [40]), we tested whether MYB44 homodimers would also be formed *in planta*. Two systems, Agrobacterium-mediated transformation of tobacco leaves and PEG-mediated transformation of Arabidopsis mesophyll protoplasts, were employed for transgene expression. The DNA constructs used in both approaches encode protein fusions of putative partners to the C (“YC”)- or N (“YN”)-terminal half of YFP. Indeed, MYB44 YN/ MYB44-YC co-expression induced fluorescence in tobacco leaf tissue (**figure 5A**). MYB44-YC/MYB44-YN-derived fluorescence was confined to the nucleus. Co-expression of MYB44 BiFC constructs in Arabidopsis mesophyll protoplasts provided further evidence for the existence of nuclear MYB44 / MYB44 aggregates (**figure 5B**). Interestingly, similar aggregates exist between MYB44 and MYB77 (**figure S4**).

**Figure 6:**
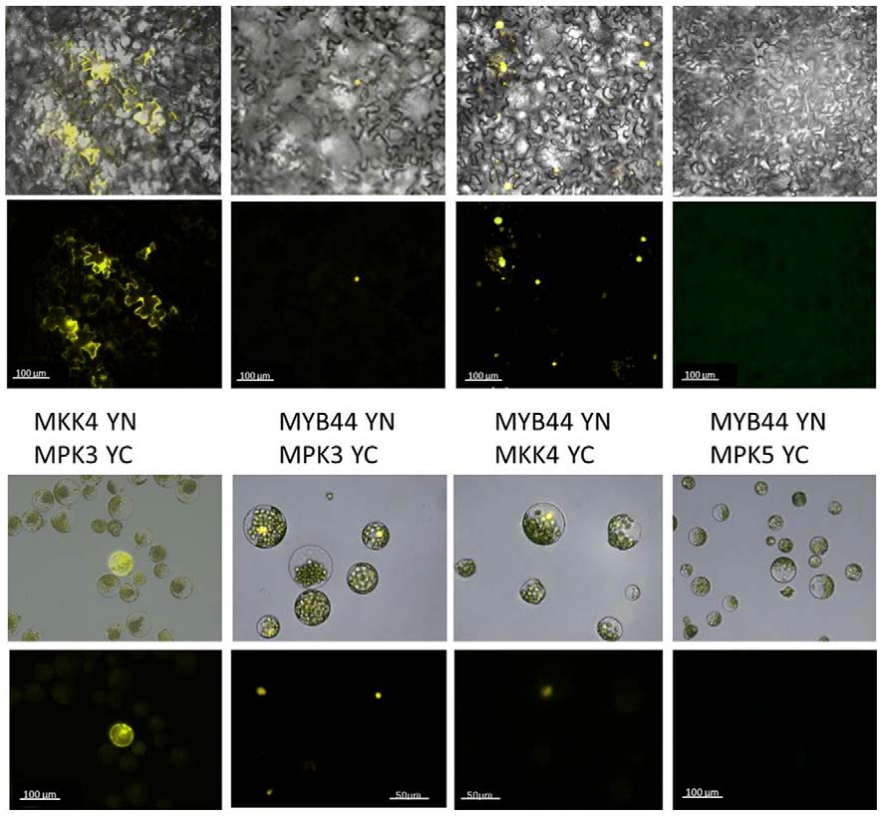
MYB44, MPK3 and MKK4 interaction *in vivo.* A) Tobacco leaves were infiltrated with Agrobacteria carrying DNA constructs of the N- or C-terminal YFP region fused to MYB44, MPK3 or MKK4 and analysed after 5d by fluorescence microscopy. B) Arabidopis mesophyll protoplasts were transformed with the above constructs and analysed 16 h post-transformation by fluorescence microscopy. top line: brightfield/UV overlay; bottom line: UV image

Next, we tested for MYB44 / MPK3 interaction *in vivo*, using a BiFC approach. Reconstituted YFP fluorescence was observed in tobacco leaves co-transformed with MYB44-YN / MPK3-YC (**figure 6A**), but not in the corresponding controls ([MPK3-YC / free -YN] or [MYB44 YN and free -YC], not shown). Furthermore, MYB44/MPK3 interaction appeared to be limited to the nucleus. Similar results were obtained from BiFC studies in Arabidopsis protoplasts upon co-transformation with the above-described DNA constructs (**figure 6B**). Reciprocal BiFC analyses (i.e. of protoplasts co-transformed with MYB44-YC/MPK3-YN) further support the existence and nuclear distribution of MYB44 / MPK3 complexes (**figure S5**). To assess whether interaction with MYB44 was a general feature of MAPKs, a distinct MAPK unrelated to MKK4 signalling, MPK5, was tested in parallel. MYB44-YN / MPK5-YC co-transformation into tobacco leaves or Arabidopsis protoplasts did not give rise to detectable fluorescence. However, reconstituted fluorescence was found in the positive control, i.e. cells co-expressing MPK3-YC and its known partner MKK4-YN, demonstrating functionality of our experimental setup. A similar nuclear signal distribution was detected, when testing MYB44 and MKK4 in BiFC assays (**figure 6A,B**), substantiating the results from Y2H experiments. In contrast, when testing for interaction between MPK3 and MKK4 we found a nucleo-cytoplasmic distribution in tobacco leaf epidermis cells and Arabidopsis protoplasts (**figure 6A,B**).

Taken together, signal distributions that were observed in BiFC assays are consistent with the reported subcellular localization of the individual proteins [41]–[43]. Moreover our results indicate that MYB44 specifically interacts with the nuclear sub-pools of MPK3 and MKK4.

### MYB44 – DNA binding properties and localization are independent of S145 phosphorylation

In order to examine the significance of MYB44 phosphorylation for its predicted function in transcriptional control, gel retardation experiments as well as a series of in vivo studies involving transient and stable expression approaches were conducted.

The affinity of transcription factors for certain DNA elements is essentially determined by their DNA binding domains. An exchange of crucial residues within that domain can completely disrupt DNA binding or alter the proteińs preference for a certain motif. *In vitro* studies with bZIP proteins in which serine residues within the bZIP domain had been systematically exchanged suggest that phosphorylation in the DNA-binding domain at selective positions could be a means of fine-tuning transcription factor activity [44]. Modifications *outside* the DNA-binding domain can also alter DNA binding properties, e.g. if protein folding and/or dimerization are affected.

The DNA consensus sequence recognized by plant MYB proteins (CCT/AACC) [45] differs substantially from that bound by animal MYB proteins(C/TAACGG) [45], [46]. MYB44 binds to the typical plant MYB binding site, MBSII from Petunia, but it also has affinity to the animal-like MBSI motif [47]. This might be due to specific features in the R2R3 recognition helices: The R2 helix is more plant-like; while the R3 helix is more animal-like [47].

**Figure 7:**
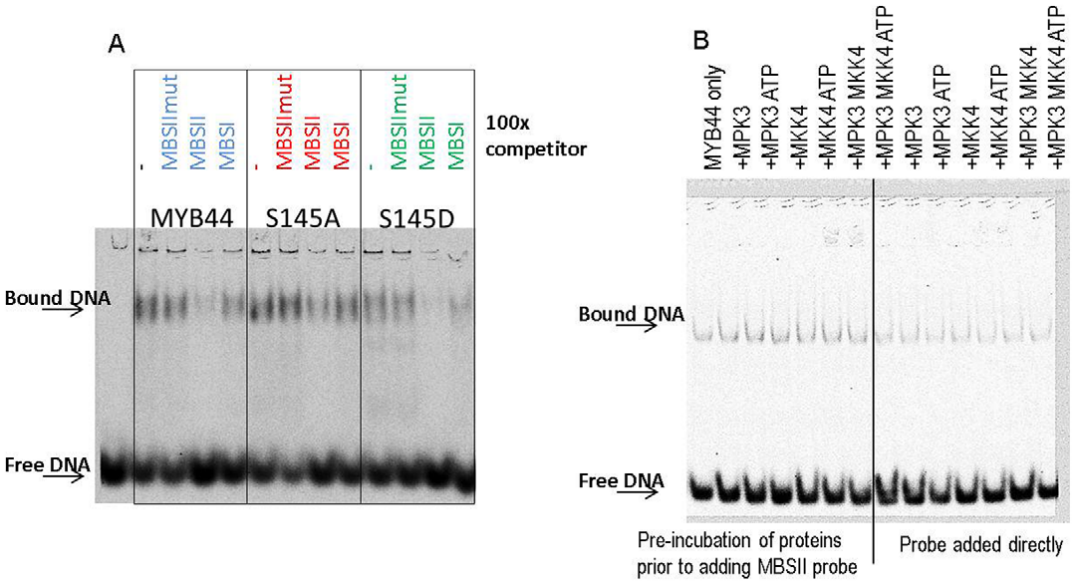
Electrophoretic mobility shift assays (EMSA). A) MYB44 – DNA binding properties are independent of S145 phosphorylation. 5′-labelled MBSII was incubated with recombinant, purified, non-tagged MYB44 or its (de)phosphomimetic variants, in the presence or absence of non-labelled competitor DNA. After 30 min incubation at room temperature, samples were resolved on a 0.5xTAE, 5% polyacrylamide gel. The experiment was repeated 5 times with similar results. B) Presence of MPK3 or MKK4 does not alter MYB44-DNA binding. EMSAs with recombinant MYB44 and labelled MBSII DNA were conducted in the presence or absence of MPK3 and/or MKK4; with and without ATP. All reagents were incubated concurrently (right), or MBSII was added after preincubation of the proteins in EMSA buffer.

To assess whether the DNA binding behaviour of MYB44 was (S145)-phosphorylation-dependent, electrophoretic mobility shift assays (EMSA) were conducted (**figure 7**). MBSII and MBSI sequences are based on Kirik et al., [47]. Recombinant (purified, tag-free) MYB44 wild type, S145A and S145D proteins were incubated with 5′-IRDye-labelled MBSII double-stranded DNA (5′-AGCTTCAAAAGTTAGTTACG-3′) in the presence or absence of a 100-fold excess of non-labelled competitor DNA. In the absence of competitors, a clear shift indicating MYB44-DNA binding was observed **(** **figure 7A**, lane 2). Adding the non-labelled competitor MBSIImut (5′-AGCTTCAAAAGccAGccACG-3′) had no discernible effect (lane 3). However, non-labelled MBSII significantly blocked MYB44 binding to the probe (lane 4). An excess of MBSI (5′-AGCTTAATGTGTGTCAGTTAGGGTGTCTCG-3′) also reduced probe binding, although less severely than the specific competitor MBSII (lane 5). Thus, MYB44 specifically binds to MBS-containing elements, with a clear preference for the plant-like MBSII type. There were no discernible differences in terms of DNA affinity or specificity between MYB44 wild type and the S145A or S145D variants (lanes 6–13). Hence, these properties of the MYB44 protein appear to be S145 phosphorylation-independent.

BiFC studies had revealed an interaction of MYB44 with MPK3, and also with MKK4, in the nucleus (**figure 6**). Theoretically, these proteins might reside in a common complex with MYB44-bound DNA. Examples of nucleoprotein complexes containing MAPKs are known from yeast [48]. Using EMSA, we tested whether the presence of MPK3 or MKK4 would alter the DNA binding characteristics of MYB44. There were no discernible differences between samples containing labelled MBSII and MYB44 alone, or MYB44 plus MPK3 and/or MKK4. Also, pre-incubation of the proteins prior to adding MBSII and/or the addition of ATP plus MPK3 (to produce phosphorylated MYB44) had no visible effect **(** **figure 7B**). Therefore, at least *in vitro*, MPK3 or MKK4 are apparently not contained in a common MYB44 – DNA complex, nor do these kinases assist or prevent MYB44 – DNA binding.

**Figure 8:**
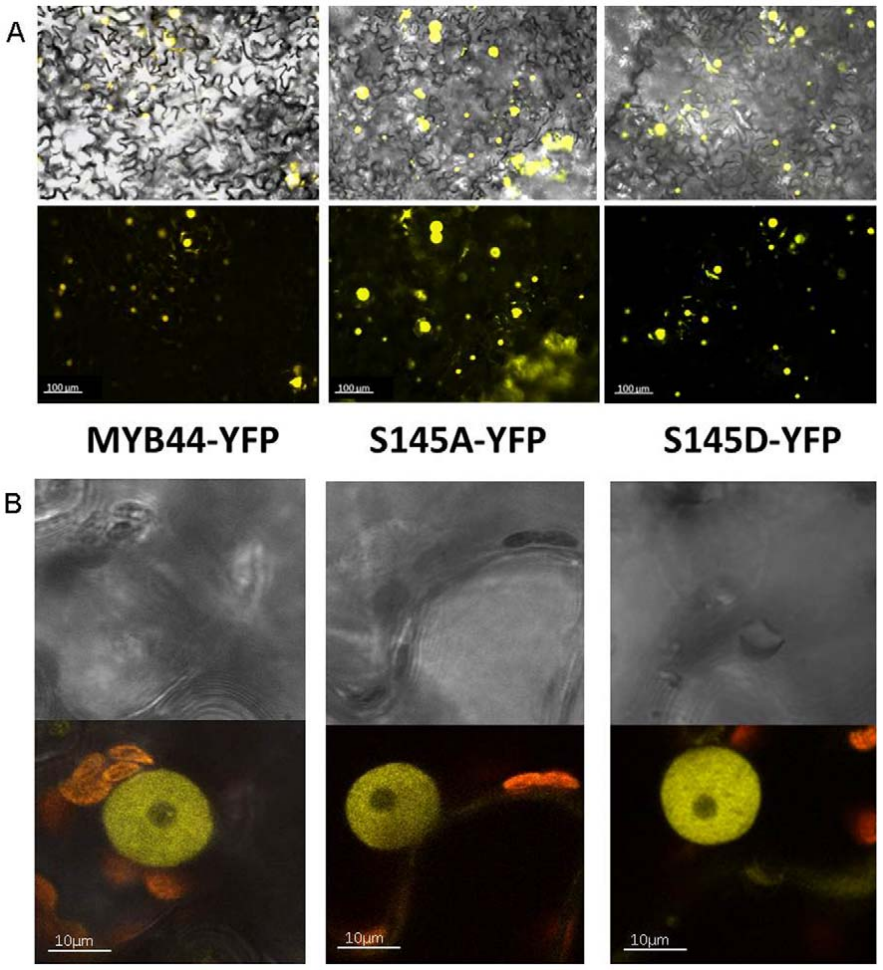
Subcellular localisation of MYB44 and phospho variants. A) Tobacco leaves were infiltrated with Agrobacteria carrying DNA constructs for the expression of MYB44 or its constitutively (non) phosphorylated variants S145A and S145D. YFP fluorescence of fusion proteins was documented 5d post-infiltration. top line: brightfield/UV overlay; bottom line: UV image B) Transient expression of above constructs in Arabidopsis mesophyll protoplasts, monitored by confocal microscopy. top: brightfield; bottom UV image

Transient ectopic expression in tobacco leaves was employed to investigate possible effects of phosphorylation on MYB44 subcellular localisation. The DNA constructs tested encoded for MYB44, MYB44 S145A and MYB44 S145D fusions to full-length yellow fluorescent protein. Overlapping with the specific distribution of MYB44 homodimers (**figure 5**), MYB44-YFP was detected exclusively in the nucleus (**figure 8**). The subcellular sorting of MYB44 is likely due to the presence of an arginine/lysine-enriched peptide region, representing a putative nuclear localisation signal (NLS). This NLS lies in the N-terminal part of the protein. Similar to MYB44-YFP, S145A and S145D-derived fluorescence signals were present only in the nucleus (**figure 8**).

**Figure 9:**
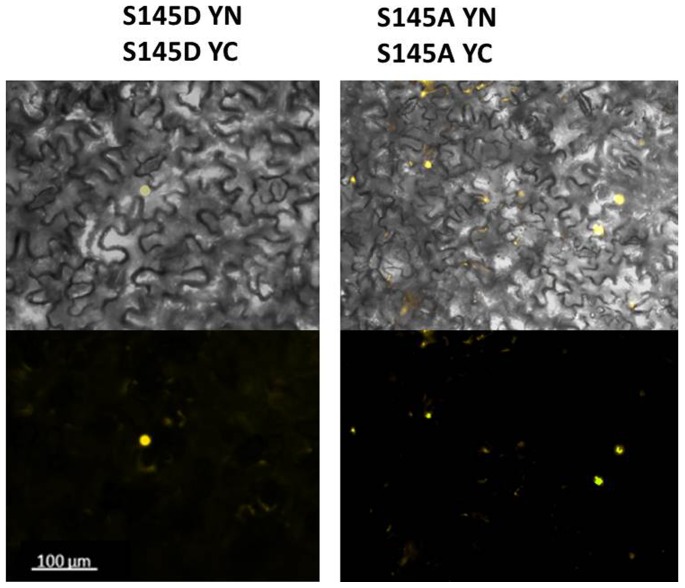
MYB44 phosphorylation at S145 does not affect formation and localisation of homodimers. Tobacco leaves were infiltrated with Agrobacteria carrying (de)phosphomimetic MYB44-split YFP constructs and analysed as described in figure 5. top line: brightfield/UV overlay; bottom line: UV image

To investigate possible effects of S145 modification on homodimerisation, MYB44 (de)phosphomimetic variants were examined by BiFC. Cells co-expressing S145A-YN/S145A-YC or S145D-YN/S145D-YC showed reconstituted fluorescence, indicative of nuclear homospecific protein complexes (**figure 9**). In conclusion, MYB44 nuclear import and/or retention as well as homodimerisation properties seem independent of S145 phosphorylation.

### MYB44 phosphorylation regulates osmotic stress tolerance

**Figure 10:**
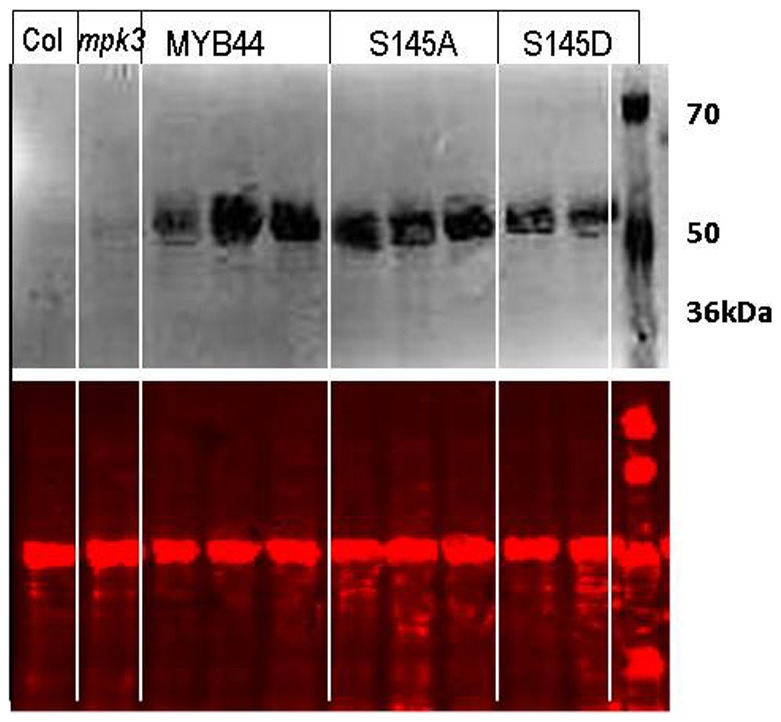
Ectopic expression of MYB44 and S145 variants in stable transgenic Arabidopsis plants. Protein extracts of 8-d-old seedlings were separated by SDS-PAGE. Transgene expression in MYB44 transgenic lines (2-3 independent lines shown per variant) was visualised by immunodetection, using rabbit anti-myc and IRDye800CW-coupled donkey-anti-rabbit (NEB Biolabs) as primary and secondary antibodies, respectively. Membranes were scanned with a LiCCOR Odyssee machine to detect bound antibody (800 nm channel, top) and to document equal sample loading (700 nm channel, bottom).

MYB44 overexpression in Arabidopsis has been reported to improve stress tolerance towards osmotic (NaCl) and drought stress [42]. Moreover, a recent study bridged MKK4 to the MPK3-mediated NaCl response [49]. As was revealed by in-gel-kinase assays, MKK4 regulates the activity of MPK3 upon NaCl exposure. *mkk4* mutants, which are impaired in MPK3 activation, have a diminished stress tolerance, whereas MPK3 hyperactivation through MKK4 overexpression has the opposite effect [49] . These observations might suggest that MYB44 overexpression-conferred stress tolerance is directly related to MKK4-MPK3 signalling and –presumably- phosphorylation-dependent. To test this hypothesis, homozygous Arabidopsis plants overexpressing *MYB44* or its (de)phosphomimetic variants, *S145A and S145D*, respectively, were generated. Transgene expression was confirmed by immunoblot analysis with an antibody directed against the C-terminal myc tag (**figure 10**). A single specific band was detected at approximately 50 kDa, which is close to the expected size (44.9 kDa). The migration of MYB44wt-, S145A- and S145D-myc fusion proteins was comparable.

Under control conditions, i.e. germination on ½ MS agar and transfer onto soil at 14 DAG, no significant differences between wild type and transgenic plants were observed. Transgene expression had no discernible effect on germination, development, seed yield or fertility, either.

**Figure 11:**
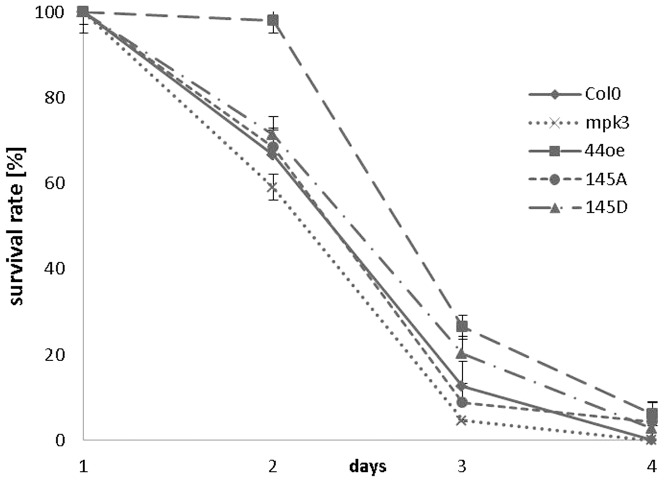
Seedling survival under salt stress. Three-day-old seedlings grown under normal conditions were transferred onto growth medium supplemented with 150 mM NaCl, and survival was monitored over a 4-day period. About 50 seedlings of three independent lines each were tested. The experiment was repeated three times with similar results.

For each MYB44 variant, three independent lines were included in subsequent stress tolerance tests. Three-day-old seedlings grown under normal conditions were transferred onto growth medium supplemented with 150 mM NaCl, and survival was monitored over a four-day period (**figure 11**). 2 days after treatment, survival in Col-0 seedlings had strongly declined (67%), and was further reduced at day 3 (12.5%) to 0% at day 4. In contrast, *MYB44* overexpressing seedlings exhibited delayed and reduced stress responsiveness (survival 2d 98%, 3d 26%, 4d 6%). These findings substantiate the already reported enhanced salt tolerance conferred by *MYB44* overexpression [42]. Congruent with the implication of the MKK4 - MPK3 module as positive regulator in the osmotic stress response [49], sensitivity was enhanced in *mpk3* mutants (survival 2d 59%, 3d 4.5%, 4d 0%). Interestingly, *MYB44S145A* overexpression also negatively affected stress survival (2d 68%, 3d 8.7%, 4d 4.7%), compared to Col-0. In fact, survival graphs of *S145A* seedlings resemble those of *mpk3*.

Regarding the phosphomimetic variant, *S145D* survival was better (2d 71%, 3d 20%, 4d 3%) than in Col-0, but lower than in *MYB44wt*. This indicates that the S145D variant cannot fully replace MYB44wt as positive regulator of salt stress signalling. Comparison of stress survival in MYB44wt, S145D and S145A lines emphasizes the importance of that particular serine residue and indicates S145 phosphorylation to be a prerequisite for MYB44-mediated salt tolerance. Consistently, repeats of the salt stress assay with additional independent transgenic lines, disclosed the same trend of tolerance levels (highest>lowest) of MYB44wt > S145D > Col-O > S145D (**figure S6**).

### MYB44 phosphorylation and abscisic acid

Many abiotic stresses, such as osmotic or drought stress, evoke the generation of the plant hormone abscisic acid (ABA) [50], [51]. Mutant plants affected in ABA synthesis, perception or signalling display abnormal phenotypes under abiotic stress conditions [5], [52], [53]. Importantly, MPK3 is activated by diverse abiotic stresses that are related to ABA signalling, and also upon exogenous application of this hormone [54], [55]. Besides regulating abiotic stress responses, ABA also acts in the promotion of seed dormancy and inhibition of seed germination [56]. *MYB44* overexpression was shown to enhance sensitivity to ABA-induced post-germination growth arrest [42]. However, the cellular/molecular processes underlying MYB44-mediated stress tolerance is still unknown. Based on the altered osmotic stress tolerance phenotypes found in MYB44, S145A and S145D lines and the implication of MPK3 in ABA-mediated responses, we suspected a relation between ABA-induced, MPK3-mediated processes and MYB44 phosphorylation.

**Figure 12:**
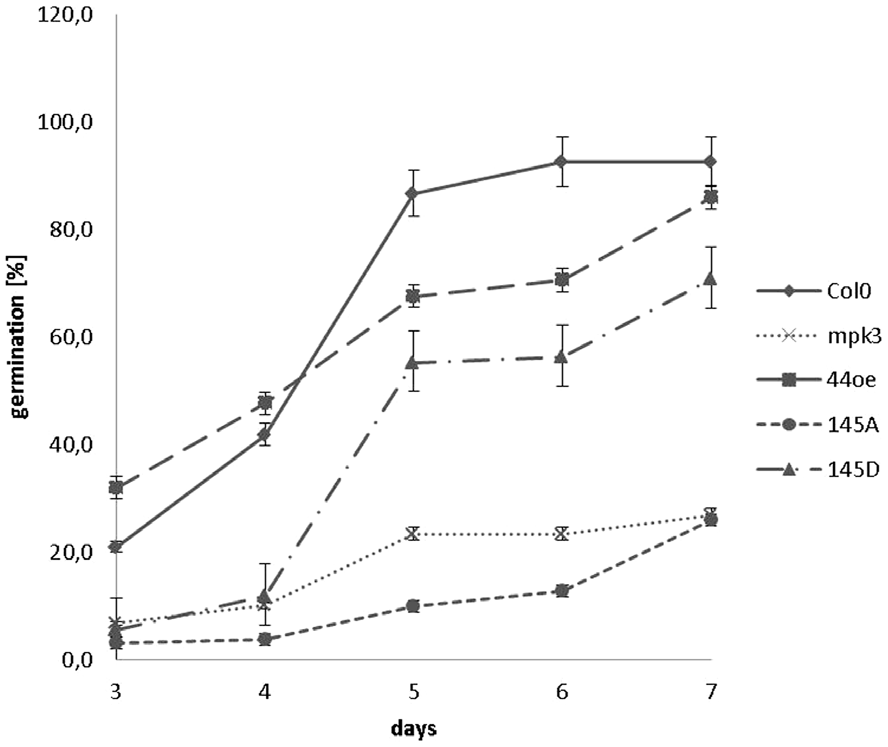
Inhibition of germination by ABA. Seeds were germinated on MS agar with or without 1µM ABA for 7d. About 50 seedlings of three independent lines each were tested. Without ABA, germination rate of all lines was 100%. The experiment was repeated three times with similar results.

To test this hypothesis, Col-0, *mpk3*, MYB44, Ser145A and S145D overexpressing lines were sown on medium supplemented with 1µM ABA, and germination was monitored over a 7-day period. On control medium lacking the hormone, seeds of all plant lines germinated equally well within two days. However, significant differences were observed in the presence of ABA. Compared to Col-0 wild type, germination in all tested lines was reduced, with *mpk3* and S145A overexpressors showing the highest sensitivity (**figure 12**). The hypersensitive phenotype of *mpk3* mutants conforms to a recent study by [41], who found Arabidopsis *pp2c5* mutants to be insensitive to germination inhibition by ABA. These mutants are deficient in the MAPK-inactivating phosphatase PP2C5 and exhibit ABA-induced MPK3 hyper-activation [41] . Accordingly, *pp2c5* and *mpk3* have opposite germination potential in the presence of ABA.

Congruent with published data [42], we found *MYB44wt* overexpression to confer enhanced ABA sensitivity. Compared to MYB44wt, germination frequency of *S145D* lines was reduced up to day four, but reached similar levels later on. In contrast, overexpression of the non-phosphorylatable S145A variant drastically reduced germination through-out the study period. Interestingly, germination graphs of S145A and *mpk3* largely overlapped - a similarity that we had noted already in the osmotic tolerance test (**figure 11**). The differences in ABA sensitivity between S145A vs. MYB44wt type or S145D lines reiterates the importance of phosphorylation at this single residue, Ser145, to MYB44 function.

## Discussion

We report the identification and characterisation of MYB44, an *in vitro* and *in vivo* interacting partner of the MAPK cascade components MKK4 and MPK3. Our study provides the first evidence of a direct link between MYB proteins and MAPKKs.

As documented by Y2H and BiFC experiments, MYB44 is an auto-interacting protein. It exclusively locates to the nucleus, where it also engages in interspecific complexes with a closely related member of the MYB S22 subfamily, MYB77. While MKK4 / MPK3 interaction occurs both in the cytoplasm and nucleus, MYB44 / MKK4 and MYB44 / MPK3 complexes are strictly nuclear (figures 5 and S6). The irreversibility of the re-established fluorophore complex in BiFC assays prevents the analysis of dynamic interactions [57]. Alternative approaches will have to be taken to reveal the kinetics of complex formation. Results from BiFC studies in cells coexpressing MYB44 and MPK5 imply that the ability to bind to MYB44 is not a general feature of MAPKs. Importantly however, MPK6, the closest homolog of MPK3, was tested positive for interaction with MYB44, both in Arabidopsis protoplasts and tobacco cells (**figure S7**). Similar to MPK3, MPK6 binds to MYB44 exclusively in the nucleus, while MPK6 / MKK4 complexes show a nucleo-cytoplasmic distribution (**figure S7**). MKK4 is a well-documented upstream regulator of MPK3 and MPK6, but not of MPK5 [58]. A scaffolding role of MYB44 in the formation of MKK4 / MPK3 and MKK4 / MPK6 complexes would contribute to specificity in MAPK cascade signalling. Whether MPK3 and MPK6 have redundant, partially overlapping, or specific functions in regulating MYB44, depending on the type of stress, remains an open question. In our study we focused on the relation of MYB44 and MPK3.

MYB44 is phosphorylated by MPK3, and we could locate the phosphorylation site to a single residue, Ser145. The faint band seen by *in vitro* kinase assay (**figure 4**) in samples of S145A and [S142A/1455A] likely arises from a minor, presumably nonspecific phosphorylation at S10 and/or S53, as it is absent in samples of S145D or S142D/S145D. A possible explanation is that, if the true target site, i.e. S145 is blocked (S145A), MPK3 yet “tries” to transfer a phosphate residue to MYB44. In contrast, MYB44 S145D or [S142D/145D] proteins, for which no signal was detected, “suggest the kinase” that it has performed its function, i.e. phosphorylation, on them already. Whether any of the three remaining Ser-Pro motifs, that match the MAPK target consensus (S/T-P), are targeted by other members of the Arabidopsis MAPK family is an open question. Our single-site location of MPK3 phosphorylation in MYB44 partially contradicts a very recent report. Nguyen et al. [59] analysed recombinant GST-MYB44 fusion proteins *after* in vitro phosphorylation via MALDI-TOF gas chromatography and concluded that both S145 *and* S53 are MAPK-targeted residues. Congruent with our observations, *in vitro* kinase assays [59] revealed a drastic reduction of phosphorylation signals upon Ser145 exchange to alanine, but in fact a similarly weak signal remained in the (S53A/S145A) double-mutated version– which in our eyes does not support the concept of S53 phosphorylation. Compared to control plants, overexpression of MYB44-S53A/S145A in Arabidopsis did not alter ABA sensitivity [59] . We think that the S53A mutation in the N-terminal DNA-binding domain interferes with the formation of the R2/R3 helices and renders MYB44 non-functional. The authors did not include single-mutated MYB44 variants or further functional assays in their study.

We investigated the possible impact of Ser145 phosphorylation on MYB44 function. The replacement of Ser145 by amino acids mimicking a constitutively nonphosphorylated (S145A) or phosphomimetic state (S145D) had no discernible effect on the proteińs subcellular distribution (**figure 8**). Possibly, recognition of the NLS by the nuclear import machinery remains unaffected by modifications at the rather distantly-located S145. Thus, unlike the MPK3-regulated bZIP transcription factor VIP1 [18], nuclear localisation of MYB44 appears to be regulated in a phosphorylation-independent manner. The capacity of MYB44 to form intraspecific complexes also is apparently maintained in S145A and S145D (**figure 9**).

We further examined the DNA binding properties of MYB44 and its S145 variants. Deletion of the C-terminal region (residues 213 onwards) was shown to enhance MYB44 - DNA binding [47], suggesting that protein features outside the R2R3 domain (spanning residues 6 – 103, **figure 2**) might contribute to the proteińs DNA affinity.

In gel retardation assays we observed similar DNA element affinity and specificity of MYB44 and its (de)phosphomimetic variants (**figure 7A**), implying that MYB44 – DNA binding is S145 phosphorylation independent. *In vitro* studies with typical animal and plant MYB proteins have demonstrated that the DNA-binding properties (strength and specificity) are determined by the corresponding MYB domains and not by other regions of the protein [60]. In contrast to the major deletion [47], a single amino acid exchange at S145, a position located in significant distance to the R2R3 domain, presumably does not alter the proteins overall conformation to an extent that would affect folding of the DNA-binding helices.

Consistent with S145A and S145D exchanges having no discernible effect on the DNA affinity or -specificity of MYB44, pre- or co-incubation with ATP and MPK3 (to produce phosphorylated MYB44) did also not alter these protein properties (**figure 7B**). Gel retardation experiments further suggest that, at least *in vitro*, MYB44 binds its cognate DNA motif independently of MPK3 and/or MKK4. Of course, it cannot be excluded that (labile) MKK4/MPK3-MYB44-DNA complexes are destabilised under native gel electrophoresis conditions.

Whether *in vivo*, the two functions of MYB44 - transcription factor and putative MKK4-MPK3 scaffolding protein – occur independently, synergistically or antagonistically is subject of our current research.

Stress tolerance tests with transgenic Arabidopsis plants clearly indicate that phosphorylation at S145 is highly relevant to MYB44 function. MYB44 properties other than its subcellular distribution, intra-specific complex formation or DNA binding characteristics (**figures 7** **–** **9**) are likely to be the cause for the altered stress performance exhibited by the MYB44 wild type, S145A or S145D overexpressing plant lines. Future studies involving transactivation capacity tests and transcriptome analyses of transgenic plants shall clarify this aspect. The affinity of MYB44 to other types of transcription factors may be controlled in a phosphorylation-dependent manner. Co-regulation of gene expression has been shown for MYB77/ARF(auxin response factor 7) [61].

Compared to Col-0, plants overexpressing MYB44 or its phosphomimetic variant S145D display elevated tolerance to salt stress, while S145A lines are more sensitive (**figures 11**). Osmotic stress survival in *S145D* was lower than in *MYB44wt* lines, indicating that the S145D variant cannot fully replace MYB44wt as positive regulator of salt stress signalling. We think that in treated seedlings a sub-pool of MYB44 exists in the phosphorylated form, and replacement at Ser145 by aspartate may only partially mimic a truly phosphorylated serine residue. Despite their enhanced tolerance towards osmotic shock treatment, MYB44 and S145D transgenic lines withstand this stress for a period of only few days (figure 11). The absence or low abundance of further stress-related factors may limit prolonged seedling survival. To determine precisely up to which stress intensity and at which developmental stage(s) MYB44 plays a major role, substantial additional research is needed.

Intriguingly, osmotic stress tolerance is suppressed in *mpk3* and S145A lines to similar extent. In *mpk3*, this reduced tolerance is likely due to the inability to phosphorylate proteins, including MYB44, that act downstream of MPK3 in stress signalling. The lowered tolerance in S145A overexpressing lines is indicative of a dominant-negative effect, which might by attributable to:

- S145A forming inactive complexes with endogenous MYB44, closely related MYB proteins or other types of transcription factors, e.g. bHLH proteins. In fact, the presence of nuclear MYB44 / MYB44 (**figure 5**) and MYB44 / MYB77 aggregates (**figure S4**) supports this assumption. Highly abundant S145A might subtract the pool of monomeric MYB proteins, preventing the formation of functional transcription factor complexes.
- S145A competition with endogenous MYB44 or close homologs (e.g. MYB77), and/or other stress-related substrates for MPK3 binding sites and phosphorylation.
- S145A blocking the formation of functional MKK4-MPK3 modules (subject of our future research).

The stress phenotypes caused by overexpression of MYB44wt, S145A or S145D are unlikely due to competition with endogenous MYB44, since loss of *myb44* was shown to have no obvious effect on abiotic stress performance [42]. As already noted by Jung et al [42], the strong similarity in expression profiles indicates a certain redundancy among the four members of the R2R3 MYB S22 subfamily. Moreover, microarray data indicate a cross-regulation within the S22 family: MYB44 overexpression correlates with reduced expression of the three remaining S22 members [42] . A possible approach to circumvent redundancy effects would involve overexpression of MYB44, S145A or S145D in mutants deficient in multiple members of the MYB S22 subfamily, e.g. (myb44/myb77/myb70/myb73). Whether such quadruple mutants would be viable and develop normally is not known. Phylogenetic considerations and in vitro kinase experiments (figures 2 and S3), revealing MPK3 phosphorylation of MYB77, but not of MYB70, suggest a particularly close relatedness between MYB44 and MYB77.

Interestingly, microarray data [42] point to a specific transcriptional auto-regulation within the MYB S22 subfamily. MYB70, MYB73 and MYB77 are among the (few) genes significantly repressed in MYB44 overexpressing plants. This might be a further indication of gene redundancy, but remains to be determined in further detail.

Our data strongly suggest that *in planta* interaction of MYB44 with MPK3 has an imminent effect: phosphorylation of the transcription factor. Yet, the meaning and outcome of the direct MYB44 – MKK4 aggregation remains hypothetical. “Short cuts” in MAPK(KK) signalling do exist, as exemplified by the direct phosphorylation of WRKY53 by the MAPKKK MEKK1 [20] . However, a direct MYB44 phosphorylation by the kinase MKK4 seems unlikely, since peptide motifs matching the minimal consensus of MAPKK-targeted proteins (dual phosphorylation at Thr-Asp-Tyr or Thr-Glu-Tyr) are not contained in the MYB44 sequence. Possible scenarios include:

- MYB44 acts as scaffolding protein, tethering the functional MKK4-MPK3 complex. By stabilising the MAPKK-MAPK sub-module, MYB44 would thus reinforce its phosphorylation by MPK3. Because MYB44 has a very low basal, but strongly stress-induced expression (Genevestigator), it would support MKK4-MPK3 association in a stress-dependent manner.
- MYB44 binding to MKK4 may prevent MKK4 association with other MAPKs, thereby ensuring MAPKK-MAPK specificity: Such function would not be unique to MYB44, since *myb44* mutants do not display developmental or stress-related abnormalities [42].
- By binding to MKK4, MYB44 may alter the cyto-nuclear distribution of MKK4 in favour of the nuclear pool. This might be achieved by assisting MKK4 nuclear import and/ or preventing MKK4 nuclear export. The subcellular site(s) of MKK4-mediated phosphorylation of MPK3 are unknown. According to our BiFC studies, the two proteins interact in cytoplasm and nucleus. Whether the cytoplasmic or nuclear micro-environment differentially facilitates MKK4 activity is still elusive.

Intriguingly, heterologous overexpression of the Arabidopsis *MYB44* gene confers an enhanced osmotic and drought stress tolerance to soybean [62]. This aspect raises several highly interesting questions, e.g.: Do soybean MAPK(s) recognize the heterologous substrate, or can MYB44 bind stress-related promoters independently of its phosphorylation status? Would the S145D phosphomimetic variant be even more effective? Is the tolerance-enhancing effect of MYB44 solely due to its function as transcription factor, or can it act as scaffold to assist specificity in MAPKK MAPK signalling similar to the (yet hypothetical) scenario in Arabidopsis? Can these functions be uncoupled? Analyses of MYB44wt and S145D alleles in the *mpk3* mutant background shall help to address some of these aspects. Furthermore, *mpk3* mutants may be employed for in-gel kinase assays to elucidate if MYB44 phosphorylation maintains correlation with stress exposure, and if MPK3 can be functionally replaced by MPK6 in phosphorylating MYB44 under these conditions.

In this study, possible roles of MYB44 phosphorylation in the biotic stress response were not investigated. Given the known role of MKK4-MPK3 during pathogen attack [63], and the implication of MYB44 in insect attack [64], harpin [65] and VIP1-mediated signalling [31], an altered stress performance in transgenic MYB44 versus its (de)phosphomimetic variant lines appears very likely. The challenge and chance for research and agriculture is to develop a MYB44 derivate that is functional in diverse plant species and that incorporates a maximum of its stress-tolerance-enhancing properties. Exploration of the potential biotechnological implications of the results represented here is part of our ongoing research.

## Methods

### Yeast-two-hybrid interaction assays

*S. Cerevisia* strain L40 was transformed with plamids pGAD (for activation domain fusion) and pBTM (for binding domain fusion) and selected on –Leu/-Trp medium, as described in [66].The vectors contained coding sequences of Arabidopsis MYB44, MAPKs or MAPKKs. Interaction was assessed using the liquid ß-galactosidase activity assay as described in .In the yeast-two-hybrid screen, strain L40 was cotransformed with MKK4-pBTM as bait, and an Arabiodopsis cDNA library contained in pAct-AD (Clontech) as prey. Cells were selected on –His/-Leu/-Trp medium.

### Plant Material, germination assay on ABA and osmotic stress test

Arabidopsis thaliana ecotype Columbia (Col-0) plants were routinuously grown at 20°C, 60% humidity under long-day conditions (16/8 h light/dark). To break dormancy, seeds were incubated at 4°C for 2 days.

At least 2–3 individual T3 transgenic lines were used for the stress assays. Experiments were independently repeated at least three times.

Seeds of Columbia-0, mpk3 (SALK_151594) and MYB44 transgenic lines were surface-sterilised with NaOCl/90% ethanol for 5 min and washed three times with 96% ethanol.

Surface-sterilised seeds were incubated on half-strength MS medium/0.25% sucrose / 1% plant agar with or without 1µM ABA. After stratification for 2d at 4°C, plates were transferred to an incubator (8/16 h dark/night regime, 25°C). Germination percentage was monitored after 3 to 7 days. Seeds were considered germinated after the cotelydones had emerged.

To assess osmotic stress tolerance, seeds were plated on half-strength MS medium (see above). After three days, seedlings were transferred to plates containing half-strength MS supplemented with 150 mM NaCl and grown for further 4 days. About 50 seeds were used for each treatment.

### Plasmids and mutagenesis

The full-length MYB44 coding sequence was PCR-amplified from Columbia-0 cDNA with primers introducing a 5′BspHI (gatattcatgactgataggatcaaaggtcc) and 3′NotI site (atagcggccgccgattctcccaactccaatttgac).

Mutated MYB44 variants were generated by site-directed mutagenesis using the following oligonucleotides:

S142A (fo: gggctttacatgGCcccaggaagcccaactggatc; re: cagttgggcttcctgggGCcatgtaaagcccagta)

S142D (fo: gggctttacatgGAcccaggaagcccaactggatc;re: cagttgggcttcctgggTCcatgtaaagcccagta)

S145A (fo: ccaggaGCTccaactggatctgatgtcagtgattc; re: aatcactgacatcagatccagttggagCtcctggg)

S145D (fo: ccaggaGAcccaactggatctgatgtcagtgattc; re: aatcactgacatcagatccagttgggTCtcctggg)

S142A145A (fo: gggctttacatgGCcccaggaGCcccaactggatctga; re: atccagttgggGCtcctgggGCcatgtaaagcccagta

S142D 145D (fo: gggctttacatgGAcccaggaGACccaactggatctga; re: atccagttgggtctcctgggtccatgtaaagcccagta

Plasmid integrity was confirmed by sequencing. The MYB44 (and phosho-derivatives) cassettes were subcloned as BspHI-NotI fragments into further target vectors (pTX3b and pGreen).

All constructs used for stable and transient expression in Arabidopsis and tobacco are based on the binary vector pGreen0029 [67], containing the CaMV35S promoter and nos terminator. MYC or YFP tags were inserted at the candidate gene 3′end as NotI-EagI fragments.

Plasmids for BiFC studies were generated by 3′ ligation of YFP fragments (YN, YC) to the candidate gene. YN and YC, incl. a spacer region, were PCR-amplified from pUC-SPYNE and pUC-SPYCE vectors [40] and cloned as NotI - BglII fragments. SPYc/nE_(fo:aggGcGGCcgcgccactagtggatccatcga; re: GaacgatcggggaaattcgagAtctta)

### Recombinant protein isolation and *in vitro* kinase assay

Taq-free recombinant MYB proteins were isolated using the intein-splicing system (IMPACT™ Kit, Biolabs). *Escherichia coli* strain BL-21 DE3 was transformed with vector pTX3B, containing the full-length coding sequences of MYB44 or its S142/S145 variants. Recombinant proteins were expressed as maltose binding protein -intein fusion proteins. Isolation, purification and final release of cleaved purified proteins was performed according to the manufactureŕs instructions. Induction, purification and isolation were monitored by SDS-PAGE/Coomassie staining.

GST-MPK3 was expressed in BL-21 and isolated as described previously [34].

In vitro kinase assays with purified GST-MPK3 and MYB proteins were performed according to [34] . Kinase reactions were heat-denatured and resolved by 12% SDS-PAGE and visualized by Coomassie staining. Vacuum-dried gels were exposed to X-ray film (Amersham) for 1-3 days.

### Arabidopsis protoplast isolation, transformation and subcellular localisation studies

Mesophyll protoplasts were isolated from leaves of 3 to 4-week-old Arabidopsis Columbia-0 plants. The protoplast isolation and transfection procedure was based on the method by Wu et al. [68], modified according to Pitzschke and Persak [69]. 12–16 h post-transformation, subcellular localisation studies were conducted at a UV microscope (Leica DM5500B), equipped with excitation/emission filters: BP450–450 nm/LP515 nm. Confocal images were acquired with a TCS-SP5 microscope using excitation wavelength of 488 nm (argon laser) for YFP and 594 nm for autofluorescence, as previously described [69].

### Transient expression in tobacco

*Nicotiana tobaccum*, leaves were infiltrated with agrobacteria (strain GV3101, carrying pSoup and the pGreen construct of interest) according to Hellens et al. [70]. 4–7 days post-infiltration, epidermal peels were investigated by microscopy as described above.

### Electrophoretic mobility shift assay

2µl 50 nM 5′Dy782-labelled double-stranded MBSII (5′-AGCTTCAAAAGTTAGTTACG-3′) (MWG Eurosynth) was incubated in EMSA buffer (10 mM Tris pH8.0, 5 mM MgCl2, 100 mM KCL, 0.02% Triton X-100, 0.1 mg/ml BSA, 0.25% Tween, 2.5 mM DTT, 3 ng/µl poly(dIdC)) alone or in the presence of 0.1 µg recombinant MYB44, S145A or S145D protein for 30 min at room temperature. For competition studies, samples contained a 100-fold excess of non-labelled MBSII, MBSII mut (5′-AGCTTCAAAAGccAGccACG-3′)) or MBSI (5′-AGCTTAATGTGTGTCAGTTAGGGTGTCTCG-3′). To study the effect of MPK3 and MKK4 on MYB44- DNA binding, samples were incubated with GST-MPK3 or GST-MKK4 and -/+ 0.5 mM ATP (directly, or pre-incubated 30 min, room temperature) before addition of the MBSII probe. 2µl loading dye (orange G, 50% glycerol in TE) was added, and 6 µl of the samples were separated on 5% polyacrylamide / 0.5x TAE gels (at 4°C, 1 h, 70V, dark). Gels were scanned at 800 nm using the Odyssee device (LiCOR). The experiments were repeated 5 times with similar results. Other electrophoresis conditions (0.5x TBE; tris/glycine) were also tested.

### Generation of stable transgenic Arabidopsis plants

The floral dip method [71]was applied to transform 6 to 7-week-old Arabidopsis Columbia- 0 plants with Agrobacteria strain GV3101, carrying the helper plasmid pSoup [67] and wild type or S142/S145 variants of MYB44-myc_pGreen. Transformants were selected on kanamycin and tested for transgene expression by immunoblot analysis. Progeny of plants showing a 1∶3 segregation (kanamycin resistance marker) were propagated in a homozygous state. All analyses were performed with homozygous lines.

### Plant protein extraction and immunodetection of MYB44-myc

Proteins were extracted from Arabidopsis leaves or eight-day-old seedlings as described by Brock et al. [41]. 15 µg protein (quantified by Bradford assay) were separated by SDS-PAGE and blotted on a PVDF membrane (Porablot, Roth). For immunodetection of MYB-myc fusion proteins, rabbit anti-myc and IRDye800CW-coupled donkey-anti-rabbit (NEB Biolabs) were used as primary and secondary antibodies, respectively. Membranes were scanned with a LiCOR Odyssee machine, at 800 nm for the detection of bound antibodies and at 700 nm to document equal sample loading.

### Gene accession numbers

MYB44 (At5g67300), MYB77 (At3g50060), MYB70 (At2g23290), MPK3 (At3g45640), MPK5 (At4g11330), MKK4 (At1g51660)

## Supporting Information

Figure S1 **MYB44 forms homodimers, but does not interact with MAPKs in the Y2H system.** Colorimetric assay of ß-galactosidase activity in protein extracts from yeast co-transformed with MYB44 and Arabidopsis MAPK family members. MYB44/MYB44 co-transformation served as positive control. Yeast transformed with MYB44 or representative members of Arabidopsis MAPK subfamilies in pBTM (binding domain) and pGad (activation domain)
(TIF)

Figure S2 **Pull-down of MPK3 with immobilised MYB44 protein.** Lane 1–3: Lysates from *E. coli* expressing 6xHis-tagged MYB44 (bait protein), GST (prey control) or GST-tagged MPK3 (prey protein). Lane 4–5: Proteins retained from E. Coli lysates after incubation with MYB44 immobilised to cobalt agarose. Lane 7: control: proteins retained from GST-MPK3 lysates after incubation with cobalt agarose only. Lane 6: identical to lane 3, for direct size comparison of MPK3 in lysate and pull-down. **Description pull-down assay (figure S2)**. Pull-down experiments further supported MYB44 – MPK3 interaction (figure KA_B). MYB44-6x His-tagged recombinant protein was immobilised to cobalt agarose, which was subsequently incubated with lysates of *E. coli* expressing GST or GST-MPK3. Detectable protein bands of app. 72kD and 46kD were retained from GST-MPK3, but not from GST samples (lanes 4 vs. 5). In the absence of MYB44, there was no detectable protein binding to cobalt agarose (lane 7). The sizes of pulled-down proteins (lane 5) correlate with the specific profile of induced GST-MPK3 *E. Coli* cultures (lane 6), and correspond to the expected size of the full fusion protein (46kD MPK3 + 26 kD GST) and free MPK3 (46kD; likely to arise from partial cleavage of the GST tag).
(TIF)

Figure S3 **Selectivity of MPK3 towards MYB proteins.** Purified, non-tagged MYB44, MYB77 or MYB70 recombinant protein was incubated with MPK3-GST in the presence of y^32^P-ATP. Phosphorylation was detected by autoradiography (right). Protein loading was visualised by Coomassie blue stainine (left).
(TIF)

Figure S4 **MYB77 interaction with MYB44.** Arabidopsis mesophyll protoplasts were co-transformed with BiFC plasmids and analysed as described in figure 5. Top: UV image, bottom: brightfield/UV overlay.
(TIF)

Figure S5 **MYB44/MPK3 interaction** ***in vivo.*** BiFC study in reciproke orientation (see figure 4). MYB44-YC and MPK3-YN were coexpressed in tobacco leaves (top) or Arabidopsis protoplasts (bottom). Complemented fluorescence was detected by UV microscopy as described in figure 4. Left panel: bright light, middle: UV image, right: overlay.
(TIF)

Figure S6 **Seedling survival under salt stress.** Three-day-old seedlings grown under normal conditions were transferred onto growth medium supplemented with 150mM NaCl, and survival was monitored over a 4-day period. About 50 seedlings of 4-6 independent lines each were tested. Results of two independent experiments are shown.
(TIF)

Figure S7 **MYB44 / MPK6 interaction** ***in vivo.*** A) Tobacco leaves were co-infiltrated with Agrobacteria carrying DNA constructs of the N- or C-terminal YFP region fused to MPK6 or MYB44, respectively and analysed after 5d by fluorescence microscopy. MKK4 / MPK6 co-infiltration served as positive control in these BiFC experiments. B) To observe MYB44 / MPK6 interaction in Arabidopis mesophyll cells, protoplasts were transformed with the indicated constructs and analysed 16h post-transformation by fluorescence microscopy. Left panel: bright light, middle: UV image, right: overlay.
(TIF)

Methods S1 (DOC)
