# Supplementary figures and images for: Tight Interconnection and Multi-Level Control of Arabidopsis MYB44 in MAPK Cascade Signalling
Source: PLoS One. 2013 Feb 21;8(2):e57547. doi: 10.1371/journal.pone.0057547 (PMC3578790; doi:10.1371/journal.pone.0057547)

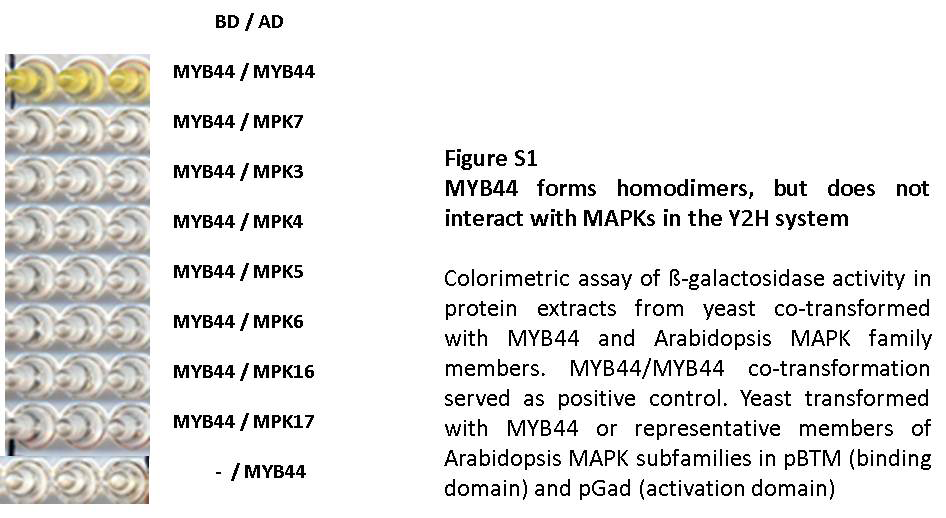

Supplement: Figure S1 — MYB44 forms homodimers, but does not interact with MAPKs in the Y2H system. Colorimetric assay of ß-galactosidase activity in protein extracts from yeast co-transformed with MYB44 and Arabidopsis MAPK family members. MYB44/MYB44 co-transformation served as positive control. Yeast transformed with MYB44 or representative members of Arabidopsis MAPK subfamilies in pBTM (binding domain) and pGad (activation domain) (TIF) [file pone.0057547.s001.tif]

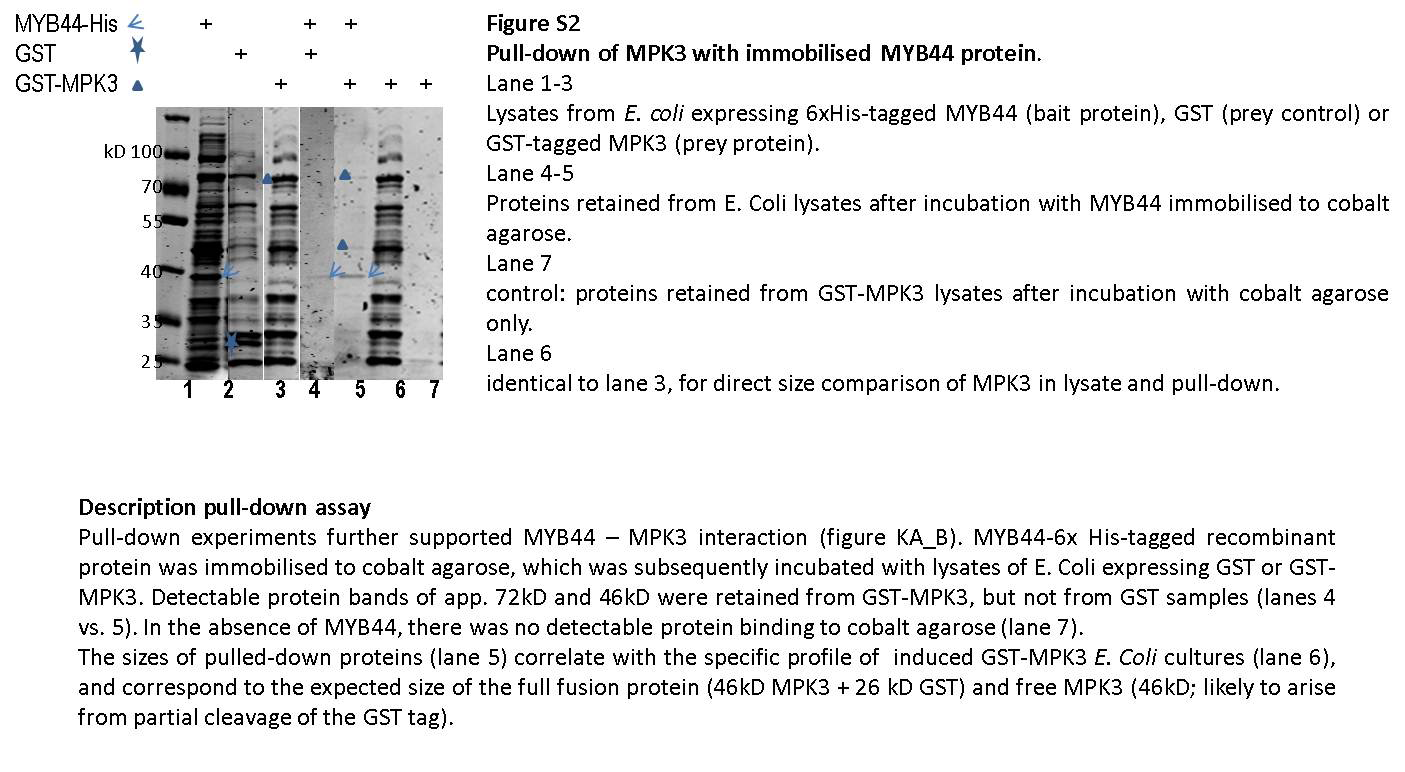

Supplement: Figure S2 — Pull-down of MPK3 with immobilised MYB44 protein. Lane 1–3: Lysates from E. coli expressing 6xHis-tagged MYB44 (bait protein), GST (prey control) or GST-tagged MPK3 (prey protein). Lane 4–5: Proteins retained from E. Coli lysates after incubation with MYB44 immobilised to cobalt agarose. Lane 7: control: proteins retained from GST-MPK3 lysates after incubation with cobalt agarose only. Lane 6: identical to lane 3, for direct size comparison of MPK3 in lysate and pull-down. Description pull-down assay (figure S2). Pull-down experiments further supported MYB44 – MPK3 interaction (figure KA_B). MYB44-6x His-tagged recombinant protein was immobilised to cobalt agarose, which was subsequently incubated with lysates of E. coli expressing GST or GST-MPK3. Detectable protein bands of app. 72kD and 46kD were retained from GST-MPK3, but not from GST samples (lanes 4 vs. 5). In the absence of MYB44, there was no detectable protein binding to cobalt agarose (lane 7). The sizes of pulled-down proteins (lane 5) correlate with the specific profile of induced GST-MPK3 E. Coli cultures (lane 6), and correspond to the expected size of the full fusion protein (46kD MPK3 + 26 kD GST) and free MPK3 (46kD; likely to arise from partial cleavage of the GST tag). (TIF) [file pone.0057547.s002.tif]

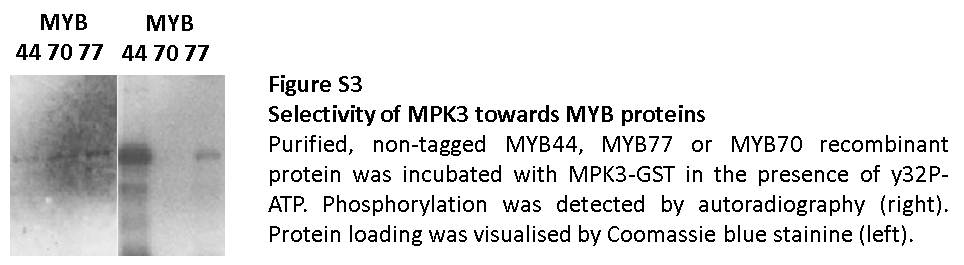

Supplement: Figure S3 — Selectivity of MPK3 towards MYB proteins. Purified, non-tagged MYB44, MYB77 or MYB70 recombinant protein was incubated with MPK3-GST in the presence of y32P-ATP. Phosphorylation was detected by autoradiography (right). Protein loading was visualised by Coomassie blue stainine (left). (TIF) [file pone.0057547.s003.tif]

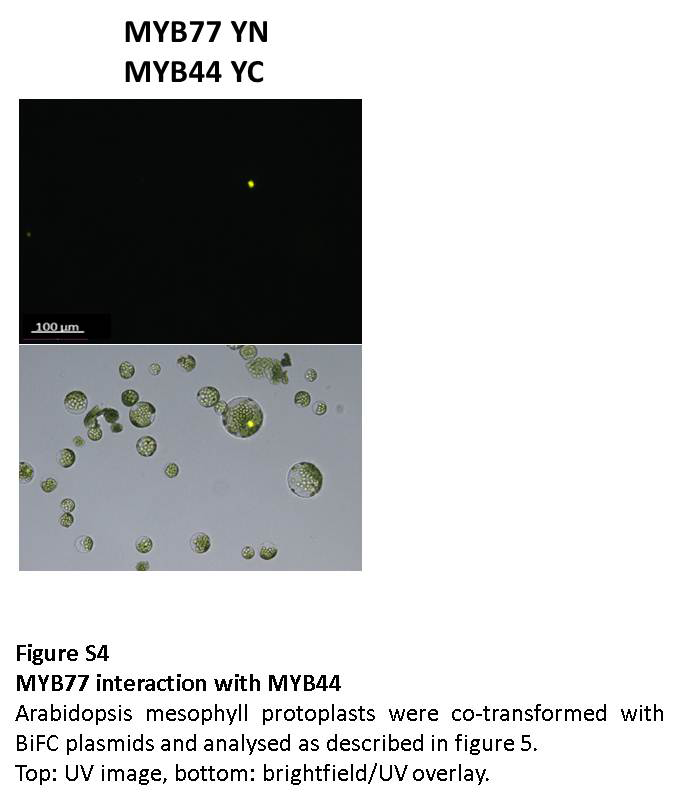

Supplement: Figure S4 — MYB77 interaction with MYB44. Arabidopsis mesophyll protoplasts were co-transformed with BiFC plasmids and analysed as described in figure 5. Top: UV image, bottom: brightfield/UV overlay. (TIF) [file pone.0057547.s004.tif]

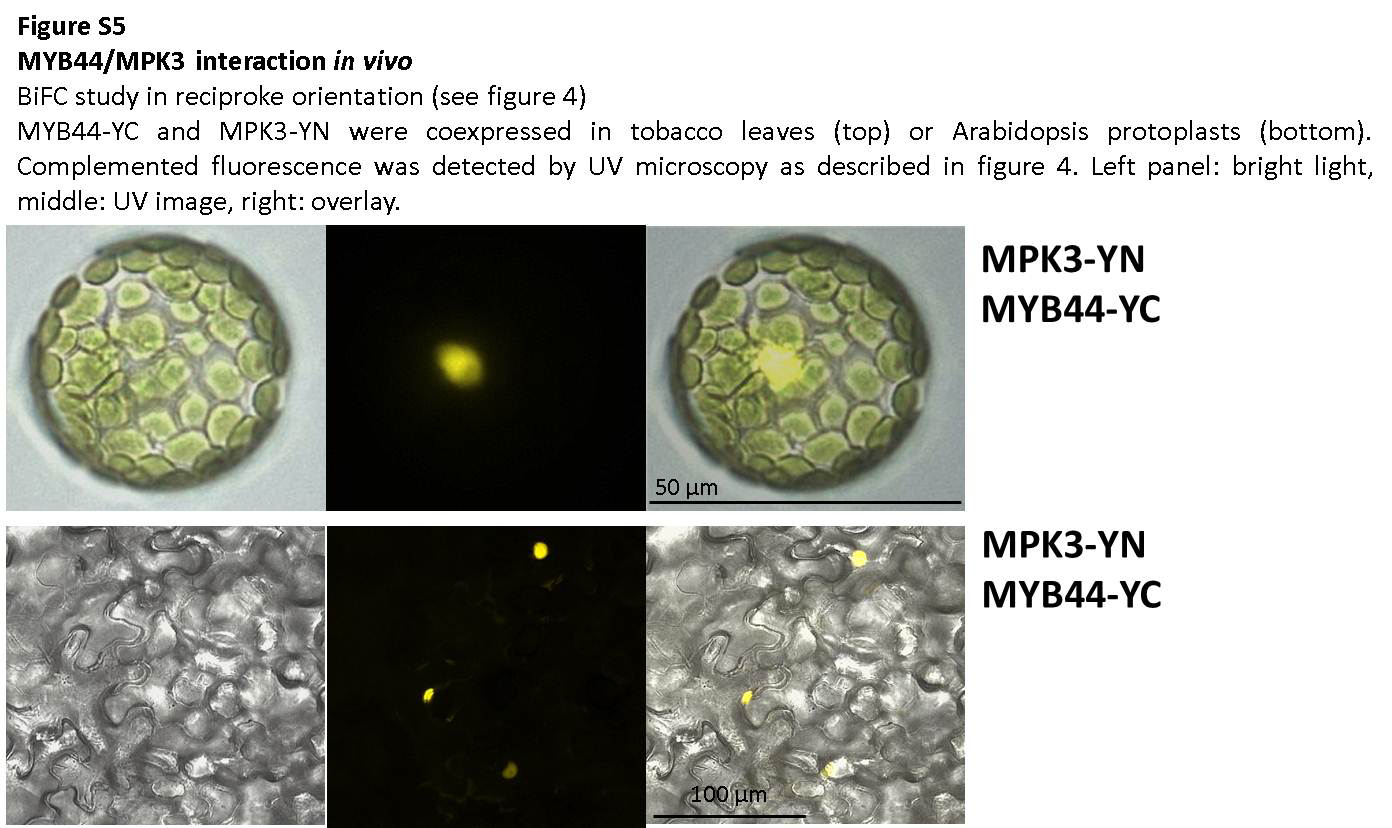

Supplement: Figure S5 — MYB44/MPK3 interaction in vivo. BiFC study in reciproke orientation (see figure 4). MYB44-YC and MPK3-YN were coexpressed in tobacco leaves (top) or Arabidopsis protoplasts (bottom). Complemented fluorescence was detected by UV microscopy as described in figure 4. Left panel: bright light, middle: UV image, right: overlay. (TIF) [file pone.0057547.s005.tif]

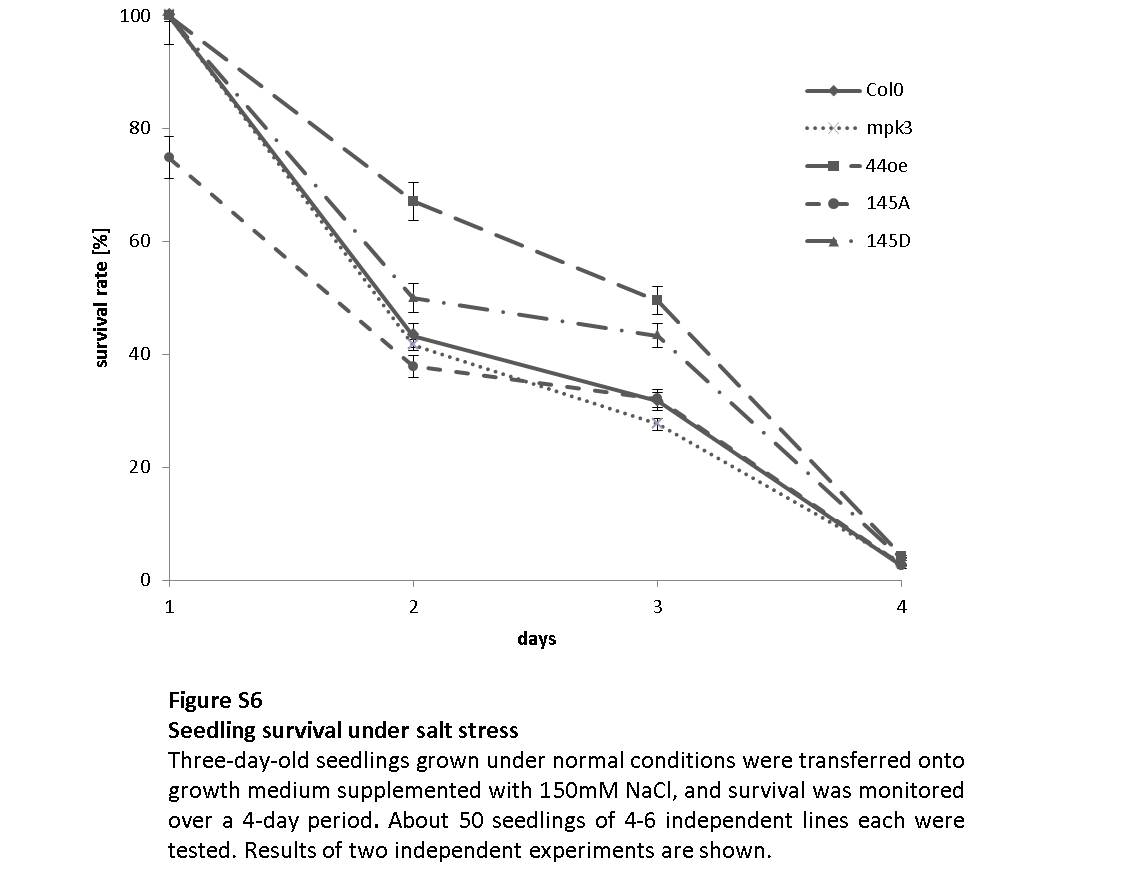

Supplement: Figure S6 — Seedling survival under salt stress. Three-day-old seedlings grown under normal conditions were transferred onto growth medium supplemented with 150mM NaCl, and survival was monitored over a 4-day period. About 50 seedlings of 4-6 independent lines each were tested. Results of two independent experiments are shown. (TIF) [file pone.0057547.s006.tif]

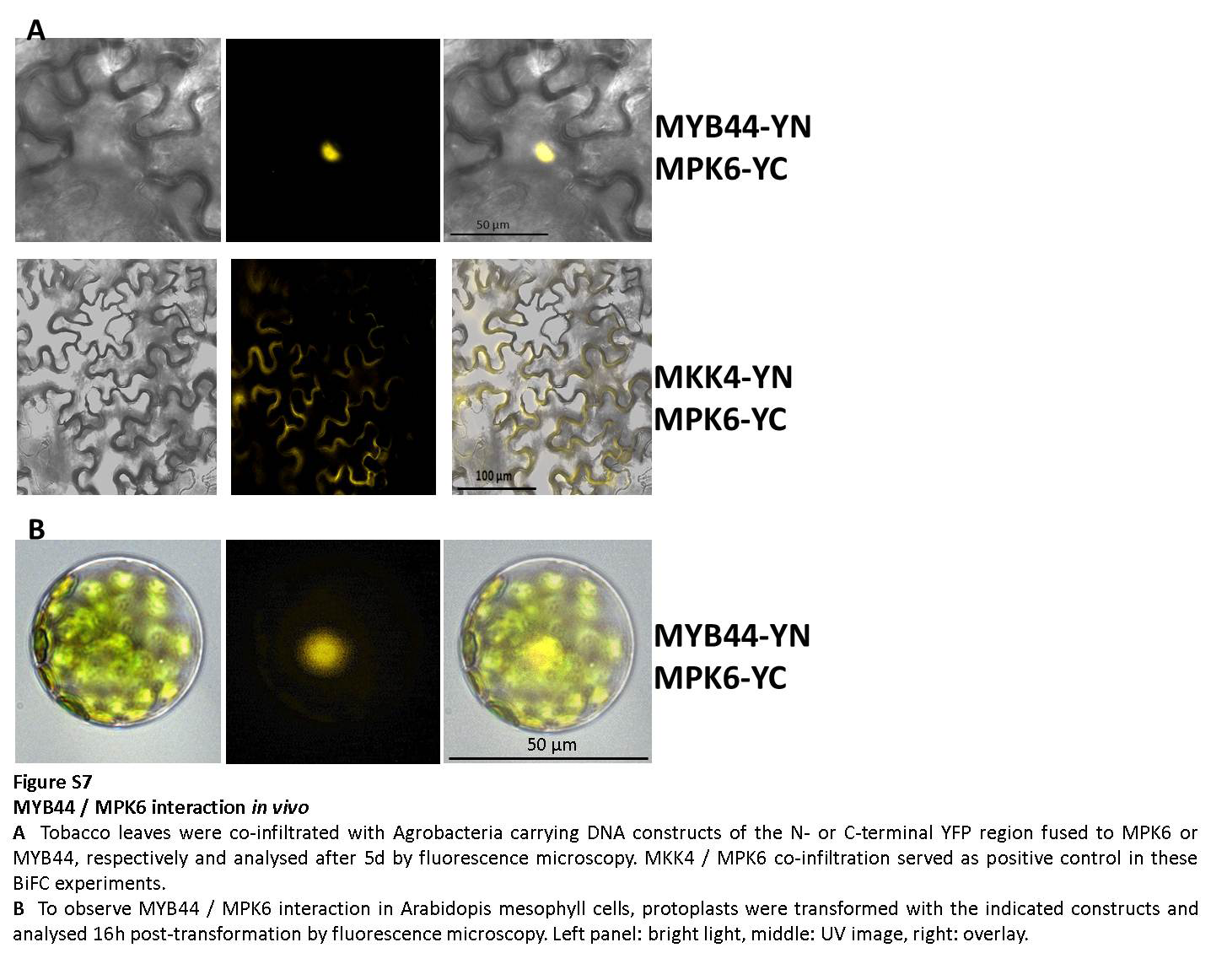

Supplement: Figure S7 — MYB44 / MPK6 interaction in vivo. A) Tobacco leaves were co-infiltrated with Agrobacteria carrying DNA constructs of the N- or C-terminal YFP region fused to MPK6 or MYB44, respectively and analysed after 5d by fluorescence microscopy. MKK4 / MPK6 co-infiltration served as positive control in these BiFC experiments. B) To observe MYB44 / MPK6 interaction in Arabidopis mesophyll cells, protoplasts were transformed with the indicated constructs and analysed 16h post-transformation by fluorescence microscopy. Left panel: bright light, middle: UV image, right: overlay. (TIF) [file pone.0057547.s007.tif]
