# Supplementary material for: Tight Interconnection and Multi-Level Control of Arabidopsis MYB44 in MAPK Cascade Signalling
Source: PLoS One. 2013 Feb 21;8(2):e57547. doi: 10.1371/journal.pone.0057547 (PMC3578790; doi:10.1371/journal.pone.0057547)
Supplement: Methods S1 — (DOC) [file pone.0057547.s008.doc]

**Supporting information**

**Methods**

***In vitro* pull-down assay**

The coding sequence of MYB44 was cloned into pET24. MYB44-6xHis, GST and GST-MPK3 were expressed in IPTG-induced Escherichia coli BL-21. Bacteria cells were sonified in 1x TBS incl. 1mM PMSF and protease inhibitors. After addition of Triton (0.1%), cells were incubated on ice for 30min. The supernatent fluid obtained after centrifugation (4°C, 10min, 12,000g) was adjusted to “P-buffer” (Tris 20mM pH7.5/ NaCl 75mM/ 0.1% Triton/ 10mM imidazol). MYB44-6x His-tagged recombinant protein was immobilised to cobalt agarose by rotating the samples at 4°C for 4h. Mock binding reactions contained P-buffer only. Cobalt agarose (with or without immobilised MYB44) was washed 5x in P-buffer and subsequently aliquoted into 3 tubes. P-buffer, or lysates of GST or GST-MPK3 were added, and samples were rotated at 4°C over night. Cobalt beads were washed 5x in P-buffer and finally resuspended in protein loading buffer. Retained proteins were heat-denatured, resolved by 10% SDS-PAGE and visualised by Coomassie blue staining.

**BiFC studies with MPK6**

The coding sequence of MYB44 in MYB44-YN and MYB44-YC constructs was replaced by MPK6. The resulting constructs, MPK6-YN and MPK6-YC, were transformed into Agrobacteria, prior to tobacco leaf infiltration, or into Arabidopsis protoplasts. Co-transformations of MPK6 with MKK4 or MYB44 were performed and analysed as described in the main manuscript file.

**Reciprocal BiFC studies with MYB44/MPK3**

The coding sequence of MYB44 in MYB44-YN was replaced by MPK3. The resulting construct, MPK3-YC was co-transformed with MYB44-YN into tobacco leaf cells as described in the main manuscript file.
